# Germination, Physiological Responses and Gene Expression of Tall Fescue (*Festuca arundinacea* Schreb.) Growing under Pb and Cd

**DOI:** 10.1371/journal.pone.0169495

**Published:** 2017-01-03

**Authors:** Yanhong Lou, Peng Zhao, Deling Wang, Erick Amombo, Xin Sun, Hui Wang, Yuping Zhuge

**Affiliations:** College of Resources and Environment, Shandong Agricultural University, Tai’an City, Shandong, P. R. China; INRA, FRANCE

## Abstract

Cadmium (Cd) and lead (Pb) are recognized as the most toxic metal ions due to their detrimental effects not only to plants, but also to humans. The objective of this study was to investigate the effects of Cd and Pb treatments on seed germination, plant growth, and physiological response in tall fescue (*Festuca arundinacea* Schreb.). We employed six treatments: CK (nutrient solution as control), T1 (1000 mg L^-1^ Pb), T2 (50 mg L^-1^ Cd), T3 (150 mg L^-1^ Cd), T4 (1000 mg L^-1^ Pb+50 mg L^-1^ Cd), T5 (1000 mg L^-1^ Pb+150 mg L^-1^ Cd). Antagonistic and synergistic actions were observed in tall fescue under Pb and Cd combined treatments. Under low Cd, plants exhibited higher relative germination rate, germ length, VSGR, catalase (CAT) and peroxidase (POD) activities. Additionally, in the shoots, the gene expression level of *Cu/Zn SOD*, *FeSOD*, *POD*, *GPX*, translocation factors, MDA, EL, and soluble protein contents were reduced under Pb stress. Conversely, under high Cd level, there was a decline in NRT, Pb content in shoots, Pb translocation factors, CAT activity; and an increase in VSGR, Pb content in roots, gene expression level of *Cu/ZnSOD* and *POD* in tall fescue exposed to Pb^2+^ regimes. On the other hand, tall fescue plants treated with low Cd exhibited lower relative germination rate, germination index, germ length, NRT, Cd content in roots. On the other hand there was higher Cd content, Cd translocation factor, CAT and POD activities, and gene expression level of *Cu/Zn SOD*, *FeSOD*, *POD*, *GPX* under Pb treatment compared with single Cd^2+^ treatment in the shoots. However, after high Cd exposure, plants displayed lower NRT, Cd content, CAT activity, and exhibited higher Cd contents, Cd translocation factor, MDA content, gene expression level of *Cu/ZnSOD* and *GPX* with the presence of Pb^2+^ relative to single Cd^2+^ treatment. These findings lead to a conclusion that the presence of low Cd level impacted positively towards tall fescue growth under Pb stress, while high level of Cd impacted negatively. In summary, antioxidant enzymes responded to Cd and Pb interaction at an early stage of exposure, and their gene expression profiles provided more details of the activation of those systems.

## Introduction

Soil contamination by heavy metals due to increased human activities including mining, industry activities, transportation, and agriculture raises major global environmental and human health concern [1]. Due to their toxicity and non- biodegradability metal ions pose a threat to plants by accumulating in edible parts which eventually could enter into the food-chain posing threat to human health [2]. Cadmium (Cd) and lead (Pb) are regarded as the most toxic environmental pollutants, as they display the most profound mobility in the soil environment [3]. It is well documented that excess Cd or Pb inhibit plant growth, and directly or indirectly interferes with their physiological processes through disrupting their metabolism [4, 5].

Since seed germination is the first physiological process to encounter abiotic stress, the ability of a seed to germinate in a medium containing metal ions would be indicative of its level of tolerance to this metal [6]. Inhibition of germination may result from the interference of metal ions with crucial enzymes. Plants subjected to high concentrations of metal ions could generate reactive oxygen species (ROS) such as O_2_^-^, O^-^ and OH^-^ [7]. Studies have rigorously documented that ROS are potentially harmful to the cell due to their oxidative damage to cellular structure and function [8]. Therefore, to alleviate this oxidative damage, plants have developed a complex antioxidative defense system, including low-molecular mass antioxidants as well as antioxidative enzymes, such as catalase (CAT), peroxidases (POD), superoxide dismutase (SOD), ascorbate peroxidase (APX) [9, 10]. SOD is one of the major O_2_^-^ scavengers for catalyzes the dismutation of O_2_^-^, resulting in molecular O_2_ and H_2_O_2_ formation [11]. However, H_2_O_2_ is also toxic to cells and has to be further detoxified by CAT and/or POD to O_2_ and H_2_O [12]. Meanwhile, another way for H_2_O_2_ detoxification is due to the oxidation and re-reduction of ascorbate and glutathione through the APX in the ascorbate-glutathione cycle [13]. The effects of Cd- and Pb- induced oxidative stress in plants have been reported via the increase or decrease in the antioxidant enzyme activities and alternations in the levels of antioxidant molecules [14, 15, 16]. Notably, maintenance of antioxidant enzyme activities were illustrated as to play important roles in scavenging metal ions induced ROS. In addition, it was suggested that heavy metal tolerance depended on the ability of plants to maintain a balance between the production of toxic oxygen derivatives and capacity of antioxidative defense systems to scavenge [10]. On the other hand, under heavy metal stress, both the transcript levels and the enzyme activities of the corresponding genes in the antioxidant systems could be induced. Tzure-Meng et al. [17] reported that there was an induction in the activities of FeSOD, APX, and Glutathione reductase (GR) in the marine macroalga *Ulva fasciata* to alleviate the oxidative damage under Cd stress.

Clear understanding of gene expression underlying the changes in antioxidant enzyme activities could provide insight into molecular adaptation of plants to heavy metal stress. Upregulated expressions were observed for GR, APX, and glutathione *S*-transferase genes induced by abiotic stress in grass pea (*Lathyrus sativus*) exposed to Pb regime [18]. Srivastava et al. [19] detected the increased expression of type-2 metallothionein, and aminocyclopropane carboxylic acid synthase/oxidase in rattlebox (*Sesbania drummondii*) subjected to Pb treatment. Liu et al. [20] reported that more than 1310 genes were affected in the expression profiles of *A*. *thaliana* in response to different concentrations (1, 10, and 100 μM) of Pb during the early stage of treatment, and most of the upregulated genes were also found under other stress. Upregulated expressions were also observed for *MnSOD*, *FeSOD*, *CytCu/ZnSOD*, *ChlCu/ZnSOD* and *POD* induced by Cd stress in perennial ryegrass [21].

Tall fescue (*Festuca arundinacea* Schreb.) is widely utilized as turf and forage in temperate regions due to its resistance to heat, drought, and wear [22]. As a potential phytoextration species, tall fescue grows well when subjected to moderate levels of Pb, and no significant difference in biomass between treatments and controls was observed [23]. Superior Cd tolerance of tall fescue was also reported by Xu and Wang [24], and tall fescue could tolerate up to 50–200 mg·kg^-1^ Cd^2+^. However, previous studies only focused on the physiological responses and gene expression in tall fescue to single metal ion, such as Cd, Pb, and Zn, and hence little is known about the physiological responses and transcription profiles of the genes coding for antioxidant enzymes in tall fescue to two metal ions or more. Herein, the objectives of this study were to investigate the physiological response and related mRNA response of tall fescue exposed to both Cd and Pb regimes, to study the relationship between plant toxicity, oxidative stress and detoxification responses, and to explore the accumulation ability.

## Materials and Methods

### Germination experiment

Seeds of tall fescue “Crossfire” were obtained from Clover Group, Beijing. Quality seeds were surface-sterilized in 0.1% potassium permanganate for 15 min, and then washed thoroughly with deionized water. Twenty seeds were uniformly placed in petri dishes (12 cm diameter) with double-layered filter paper (3 mm, whatman, Maidstone, UK) on the bottom, moistened with 10 mL Cd^2+^ or Pb^2+^ aqueous solution of various concentrations. According to the pre-experimental results, six treatments were employed: CK (nutrient solution as control), T1 (1000 mg L^-1^ Pb), T2 (50 mg L^-1^ Cd), T3 (150 mg L^-1^ Cd), T4 (1000 mg L^-1^ Pb+50 mg L^-1^ Cd), T5 (1000 mg L^-1^ Pb+150 mg L^-1^ Cd), and all Cd^2+^ and Pb^2+^ were dissolved in nutrient solution. The composition of nutrient solution were 0.5 mM NH_4_H_2_PO_4_, 0.5 mM KNO_3_, 0.5 M Ca(NO_3_)_2_•4H_2_O, 0.5 M MgSO_4_, 1.43 μM H_3_BO_3_, 0.91 μM MnCl_2_, 0.11 μM ZnSO_4_, 0.44 μM CuSO_4_, 0.01 μM H_2_MoO_4_, 20 μM Fe•EDTA. Three replicates were employed for each treatment. All petri dishes were placed randomly in growth chambers set at temperatures of 28°C/20°C (day/night). The petri dishes were covered with the lids and kept in a growth chamber for 3 days in the dark and 6 days with 16 h photo-period, with a light intensity of 240 μ mol photons m^-2^ S^-1^ and a relative humidity of 65% at 28°C/ 20°C (day/night). To maintain the same germination cultivation, we added the culture solution every two days.

### Seedling growth experiment

Fifteen seeds of tall fescue (cv. Crossfire) were selected and sown in disposable plastic cups (7.5 cm in diameter×9.0 cm deep). All cups were filled with pre-treated sand (<1 mm) and perforated at the base with an orifice of 5 mm diameter. The sand was pre-treated with 5% HCl (v/v) initially, and then rinsed with tap water followed by deionized water successively, and subsequently air dried. Filled cups were placed randomly in growth chambers with daily maximum and minimum temperature of 24°C and 20°C, the photo-period of 16-h and the photosynthetically active radiation PAR of 240 μ mol photons m^-2^ S^-1^ at the canopy level. After germination, half-strength Hoagland’s nutrient solution was added to provide nutrient and water to the seedlings by daily irrigation. After 40 days of cultivation, the plant roots were rinsed thoroughly with deionized water and transferred into 250-mL Erlenmeyer flasks filled with half-strength Hoagland’s solution for 3 days to adapt to the new environmental conditions. All plants of each flask were sealed with preservative film at the crown to prevent transpiration. In order to prevent algal growth, all flasks were wrapped with aluminum foils. The plant-flask system was weighed at every 24 h to determine water loss (i.e., transpiration rate) according to the method described by Fu et al.[25]. Plants with similar Tr were grouped into one replicate to maintain the same level within each treatment. Before heavy metal treatments, grasses were hand-clipped at 6 cm height. Tall fescue was subjected to certain Pb^2+^ or Cd^2+^ levels. Six treatments were as follows: CK: nutrient solution as control, T1: 1000 mg L^-1^ Pb, T2: 50 mg L^-1^ Cd; T3: 150 mg L^-1^ Cd, T4: 1000 mg L^-1^ Pb+50 mg L^-1^ Cd, T5: 1000 mg L^-1^ Pb+150 mg L^-1^ Cd. Pb^2+^ and Cd^2+^ were added and dissolved in half-strength Hoagland’s nutrient solution. All the treatments were arranged in a randomized, complete block design with three replicates in a growth chamber with 16 h photo-period, with a light intensity of 240 μ mol photons m^-2^ S^-1^ and a relative humidity of 65% at 28°C/ 20°C (day/night).

### Measurements

#### Seed germination

Germinated seeds with 1 mm radicle length were recorded at every 24 h, and radicle elongation was measured 9 days after incubation. Final germination percentage (TG) at the end of the experiment period was calculated according to the following formula:

$$TG=\frac{Number\;of\;ger\text{min}ated\;seeds\;at\;9\;days}{Total\;number\;of\;seeds}\times 100$$

Relative germination rate (%) = Germination rate in treatment×100 / germination rate in control.

Germination index was calculated as arithmetical sum of the total seeds germinated every day up to a period of 9 days.

Germ length was measured using ruler. Five seeds were selected and the average was used for analysis.

#### Plant growth

Vertical shoot growth rate (VSGR) was calculated according to the method described by Huang and Liu [26] with slight modifications. At the initial and ultimate of the treatment, turf canopy heights were measured four times with a ruler in each flask, and the average height was divided by 9 (days) to calculate VSGR [21]. Fresh weights for shoots and roots were also weighed separately at the end of the experiment (9d).

Plant transpiration was assessed following the procedure described by Yu et al. [27]. Water loss was determined by weighing the plant-flask system at every 24 h. To compare the effect of Cd^2+^ or Pb^2+^ on plants with different initial Tr, the normalized relative transpiration (NRT) was calculated according to the following equation described by Yu et al. [28]:

NRT(C,t)(%)=(1n)∑ni=1Ti(C,t)/Ti(C,0)(1m)∑mi=1Tj(C,t)/Tj(C,0)×100,

where *C* is concentration (mM), t is time period (days), T is absolute Tr (grams per day), i is replicate 1, 2, …, n, and j is control 1, 2, …, m. The relative Tr of controls is always set at 100%.

To determine leaf chlorophyll content, fresh leaves (0.1 g FW, cut into small pieces) was soaked in 15 mL dimethyl sulfoxide and kept in the dark for 72 h according to the method described by Yu et al. [29]. The absorption of leaf extracts at 663 and 645 nm was measured with a spectrophotometer [ultraviolet-2600; UNICO (Shanghai) Instruments, Shanghai, China].

Electrolyte leakage (EL) was used as a reliable and rapid method to assess membrane permeability [10]. Fully expanded leaves (0.1 g FW) were excised and washed three times with deionized water, then cut into ≈0.5-cm long segments and placed into 50 mL vials containing 20 mL distilled water. All vials were shaken for 24 h at room temperature. The initial conductivity (*C*_i_) of the incubation solution was measured with a conductance meter (JENCO-3173; Jeno Instruments, San Diego, CA). Subsequently, the leaves were transferred to and killed in an autoclave at 120°C for 30 min. After 24 h incubation on a shaker at room temperature, the conductance of the incubation solution with killed tissues (*C*_max_) was also determined. The relative EL was calculated using the following equation:

EL (%) = (Ci /Cmax)×100

#### Physiological measurements

About 0.3 g of fully expanded leaves were homogenized in a pre-chilled mortar and pestle with 4 mL of 50 mM ice-cold phosphate buffer (pH 7.0). Then the homogenate was centrifuged at 15,000 xg for 15 min at 4°C. The supernatant was collected for MDA content, enzyme activities, and soluble protein content determination.

The content of MDA was determined by thiobarbituric acid reaction as described by Heath and Packer [30] with slight modification. Briefly, a reaction solution was made containing 20% (v/v) trichloroacetic acid, 0.5% (v/v) thiobarbituric acid, and 1 mL of enzyme extract in 2 mL volume. The mixture solution was heated at 95°C for 30 min using water bath, and then quickly cooled in an ice-water bath. Eventually, the solution was centrifuged at 10,000 rpm for 10 min. The absorbance of the supernatant at 532 nm was recorded and corrected for unspecific turbidity by substracting the value at 600 nm. The content of MDA was calculated based on this adjusted absorbance and the extinction coefficient of 155 mM^-1^cm^-1^ [30].

To determine the soluble protein content, a reaction solution containing 70 μL of 150 mM PBS (pH 7.0), 30 μL soluble protein extract and 3 mL color reagent according to the protocol described by Bradford [31]. The absorbance of the reaction solution was measured at 595 nm after 2 min and before 1 h using a spectrophotometer (ultraviolet-2006).

The activity of superoxide distumase (SOD) was determined by monitoring its ability to inhibit the photochemical reduction of nitroblue tetrazolium chloride (NBT) [32]. The absorbance of the irradiated solution at 560 nm was recorded with a spectrophotometer (ultraviolet-2006), and one unit SOD activity was defined as the amount of enzyme required to cause 50% inhibition of the rate of NBT reduction at 560 nm.

Catalase activity (CAT) was determined using the method of Gill et al. [33] by monitoring the disappearance of H_2_O_2_ at 240 nm. One unit of CAT activity was defined as the absorbance change of 0.01 units per minute.

Peroxidase activity (POD) was determined following the method described by Polle et al. [34] with minor modifications. Absorbance change at 460 nm was recorded every 1 min within the first 3 min for calculating POD activity. One unit POD activity was defined as the absorbance change of 0.01 units per minute.

#### Cd and Pb content measurement

To determine the Cd or Pb content, the plant materials (shoots and roots) were killed at 105°C for 30 min, and then dried to constant weight at 80°C. Dry samples were ground with a mortar and pestle. About 0.5 g dry samples were ashed, and then subjected to wet digestion with a mixture of concentrated HNO_3_ and concentrated HClO_4_ at 5:1 (v/v) [3]. Finally, the digested solution was redissolved with nitric acid. The atomic absorption spectroscopy (Shimadzu, Model AA-7000, Japan) was employed to determine the Cd or Pb content [35], and the concentration of Cd or Pb was defined as the Cd or Pb content (mg) per unit plant (kg). Meanwhile, the translocation factor was calculated using the following equation: Translocation factor = metal concentration in shoots/metal concentration in roots [36].

#### Gene expression of antioxidant enzymes (RT-qPCR)

Gene expression was assessed according to the method described by Yu et al. [37]. Leaf samples were collected at 24 h after the initiation of the treatment and flash frozen in liquid nitrogen and then stored at -70°C.

About 0.1 g expanded leaves was homogenized with liquid nitrogen, and total RNA was isolated using Trizol reagent (Invitrogen, Carlsbad, CA). The RNA concentration and purity were determined, and the DNA contamination was removed. RNA (2 μg) was reversely transcribed with oligo (dT) primer using first strand cDNA synthesis kit according to the user manual (Fermentas Canada, Burlington, ON, Canada). Primers of different genes were synthesized according to the previous reports for use in Q-PCR (Table 1). The PCR amplification data were analyzed with Option Monitor version 2.03 (MJ Research).

**Table 1:** Primer sequences used for quantitative polymerase chain reaction analyses of tall fescue subjected to Cd and Pb stress.

Gene	Primer^y^	Primer sequence (5’-3’)	Product size (base pair)	Reference
*Cu/Zn SOD*	F	GACACMACAAATGGHTGCAT	221	Bian and Jiang[38]
	R	TCATCBGGATCGGCATGGACAAC		
*FeSOD*	F	TGCACTTGGTGATATTCCACTC	297	Kim et al. [39]
	R	CGAATCTCAGCATCAGGTATCA		
*POD*	F	AGGCCCAGTGCTHCAMCTTC	220	Bian and Jiang[38]
	R	TTGGTGTAGTAGGCGTTGTC		
*GPX*	F	GCCGAGTATCCGATTTTTGA	195	Byrne et al. [40]
	R	TCGATACTGAGCGGAGAGGT		

^y^F and R represent forward and reverse.

### Statistical analysis

One-way analysis of variance was conducted using SPSS (version 17.0; IBM Corp., Armonk, NY). Treatment means were separated using Fisher’s least significant difference at 0.05 significant levels.

## Results

### Seed germination

The relative germination rate, germination index, and germ length clearly revealed the inhibitory effects of Cd and Pb on germination of tall fescue (Table 2). In the presence of heavy metal, the seed germination of tall fescue was inhibited intensively except the lower Cd treatment (50 mg L^-1^ Cd^2+^). Compared with T1, relative germination rate in T4 was improved 20.8 percentage point at similar Pb^2+^ concentration. At the same time, lower relative germination rate was observed in T4 relative to T2, with the presence of Pb^2+^. Germ length in T4 was higher than T1, and lower than T3 at the same level of Pb or Cd. However, no significant difference was detected among T1, T3, and T5 in germ length and germination index.

**Table 2:** Effect of Cd or Pb on seed relative germination rate, germination index, and germ length in tall fescue.

Treatment	Relative germination rate (%)	Germination Index	Germ length (cm)
CK	100.00a	13.74a	7.12a
T1	25.00c	2.16cd	1.66d
T2	94.25a	10.20b	3.72b
T3	13.46c	1.74d	1.37d
T4	55.80b	5.12c	2.90c
T5	23.08c	2.45cd	1.35d

Data are expressed as the means of three replicates (n = 3). Means in a column followed by different lower-case letter for each measurement are significant at Fisher’s protected least significant difference test at *P* = 0.05

### Plant growth

Turf quality, NRT, VSGR, Chl a+b, and carotenoid content in tall fescue were decreased remarkably when exposed to Pb^2+^ or Cd^2+^ regimes (Table 3). Plants treated with both Cd^2+^ and Pb^2+^ displayed no significant difference in turf quality, Ch a+b, and carotenoid content relative to single Cd^2+^ or Pb^2+^ treatments at the same metal ion level. The decrement in NRT was dose-dependent under Cd stress, and the NRT in T3 was only 32.63%. NRT in T4 was lower than T2 with the presence of Pb^2+^. Notably, NRT in T5 was 21.63% lower than T1 and T3. VSGR in T4 and T5 were higher than T1 with the presence of Cd^2+^. However, plants in T4 and T5 treatments exhibited similar VSGR relative to T2 and T3.

**Table 3:** Effect of Cd or Pb on turf quality, vertical shoot growth rate (VSGR), normalized relative transpiration (NRT), biomass, Chla+b, and carotenoid content in tall fescue.

Treatment	Turf quality	NRT (%)	VSGR (cm d^-1^)	Chla+b (mg g^-1^ Fw)	Carotenoid content (mg g^-1^ Fw)
CK	8.17a	100.00a	0.87a	0.33a	4.22a
T1	6.33b	31.98c	0.23c	0.22b	2.59b
T2	6.17b	50.30b	0.54b	0.23b	2.87b
T3	6.17b	32.63c	0.51b	0.20b	2.31b
T4	6.33b	36.67c	0.44b	0.21b	2.73b
T5	6.00b	21.63d	0.41b	0.17b	2.31b

Data are expressed as the means of three replicates (n = 3). Means in a column followed by different lower-case letter for each measurement are significant at Fisher’s protected least significant difference test at *P* = 0.05

**Fig 1:**
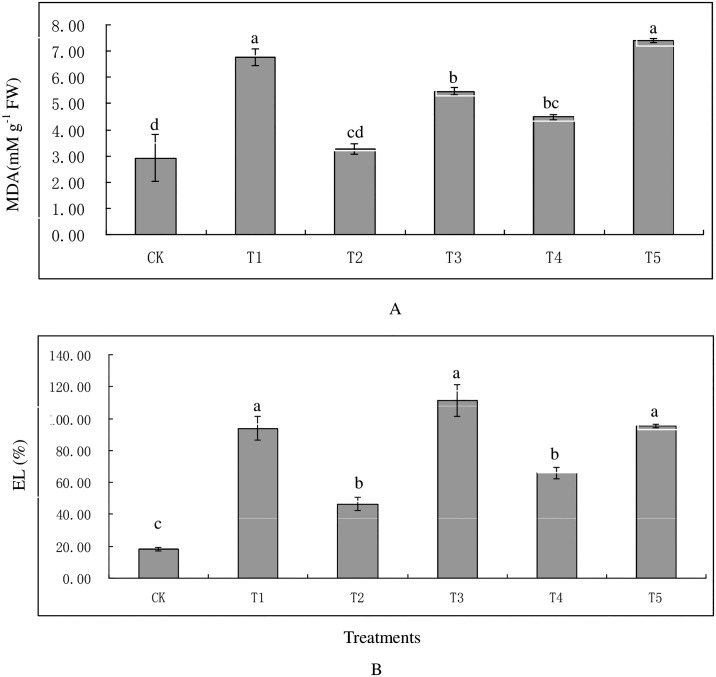
Effect of Cd and Pb on lipid peroxidation (MDA) (A) and electrolyte leakage (EL) (B) in tall fescue. Vertical bars indicate SD, and bars with same letter indicate no signigicant difference at *P* = 0.05.

**Fig 2:**
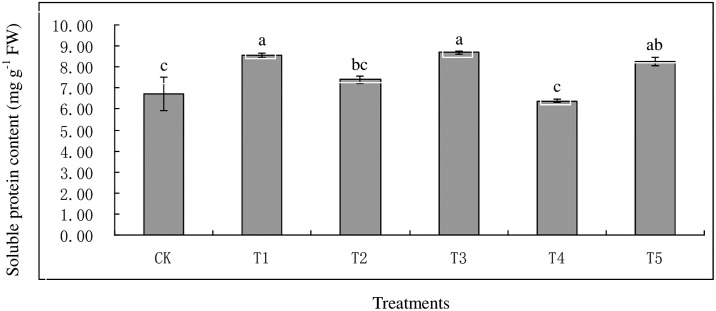
Effect of Cd and Pb on soluble protein contents on tall fescue. Vertical bars indicate SD, and bars with same letter indicate no significant difference at *P* = 0.05.

Pb^2+^ or Cd^2+^ caused a significant increase in EL content in all heavy metal treatments (Fig 1). Plants treated with T3, T5, and T1 exhibited highest EL, with 111.30%, 95.48%, and 93.80% respectively. The increment in EL was dose-dependent when plants were exposed to single Cd^2+^ regime. The EL in T4 was lower than T1 under Cd^2+^ exposure. Similar EL in T5 was observed relative to T1 and T3. Meanwhile, the MDA contents were also increased in plants exposed to Pb^2+^ or Cd^2+^ regimes except T2. Plants treated with T4 displayed lower MDA levels than T1 when exposed to Cd^2+^, while on the other hand the MDA content in plants treated with T5 was also higher than T3 in the presence of Pb^2+^. Plants treated with T1, T3, and T5 exhibited higher soluble protein contents than CK (Fig 2). Lower soluble protein content was observed in T4 relative to T1 with the presence of Cd, and no significant difference among T1, T3, and T5 at the same level of Cd or Pb.

### Cd and Pb content

Roots accumulated higher contents of Cd and Pb than shoots regardless of single metal ions or combined metal ions treatments (Table 4). The Pb contents in shoots were decreased in T4 and T5 relative to T1 with the presence of Cd^2+^, and the decrement was dose-depend on Cd^2+^. Plants treated with T5 exhibited higher Pb^2+^ content in roots than T4. Higher translocation factor was observed in T1 than T4 and T5, and the decrement of translocation factor in both Pb and Cd treatments was dose-dependent on Cd^2+^ level. Cd content in shoots was increased with the presence of Pb^2+^. However, plants treated with both Pb^2+^ and Cd^2+^ exhibited lower Cd contents in roots relative to single Cd^2+^ treatments at the same Cd^2+^ level. Higher translocation factors were assessed in both Cd and Pb treatments than single Cd^2+^ treatment, and plants treated with T4 exhibited higher translocation factor than T5.

**Table 4:** Translocation factor and Cd, Pb contents in shoots and roots of tall fescue subjected to Cd or Pb regime.

Treatment	Pb			Cd		
	Shoots (mg kg^-1^)	Roots (mg kg^-1^)	Translocatio factor	Shoots (mg kg^-1^)	Roots (mg kg^-1^)	Translocation factor
CK	0d	0c	0d	0d	0d	0d
T1	152.44Ba	160.97Ab	0.95a	0d	0d	0d
T2	0d	0c	0d	19.49Bc	250.30Aab	0.08c
T3	0d	0c	0d	32.13Bb	291.97Aa	0.11c
T4	132.43Bb	156.24Ab	0.85b	111.17Ba	181.00Ac	0.63a
T5	109.25Bc	196.40Aa	0.56c	100.23Ba	210.53Abc	0.48b

Data are expressed as the means of three replicates (n = 3). Means in a column followed by the different lower-case letter for each measurement are significant; means in a row followed the same upper-case letter for each measurement are non significant using Fisher’s protected least significant difference test at *P* = 0.05

### Activity of antioxidant enzymes

The activities of SOD and POD were increased significantly regardless of single Pb^2+^, Cd^2+^, or Pb^2+^+Cd^2+^ interaction (Table 5). However, plants treated with single or combined Pb^2+^ and Cd^2+^exhibited lower activity of CAT relative to CK. No significant difference in activity of SOD was observed between either T1 and T4 or T2 and T4 when plants exposed to the same level of Pb^2+^ or Cd^2+^ regimes. The activity of SOD in T5 was similar to T1 and T3. Plants treated with T4 exhibited higher activity of CAT than T1 or T2. However, lower activity of CAT was observed in T5 relative to T1 or T3. Similar trends of POD were detected in T4, T1 and T2. However, no significant difference was observed among T1, T3, and T5.

**Table 5:** Effect of Cd or Pb on activities of superoxide dismutase (SOD), catalase (CAT), and peroxidase isozyme (POD) in tall fescue.

Treatment	SOD (U min^-1^ mg^-1^ protein)	CAT (U min^-1^ mg^-1^ protein)	POD (U min^-1^ mg^-1^ protein)
CK	20.86e	80.54a	1365.89c
T1	29.15bc	46.78c	1888.30b
T2	26.30d	54.06c	1637.47b
T3	32.56a	47.39c	1702.66b
T4	27.40cd	65.23b	2438.92a
T5	30.92ab	29.91d	1984.24b

Data are expressed as the means of three replicates (n = 3). Means in a column followed by different lower-case letter for each measurement are significant using Fisher’s protected least significant difference test at *P* = 0.05

### Gene expression of antioxidant enzymes

**Fig 3:**
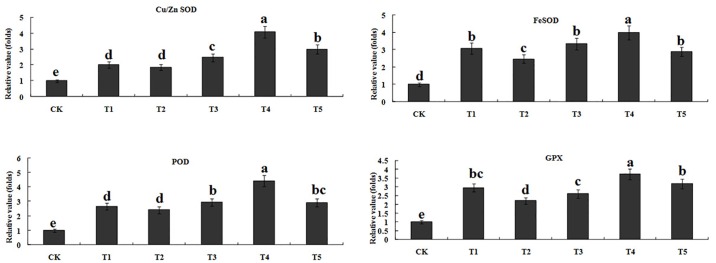
Temporal transcription changes of different types of superoxide dismutase (SOD), peroxidase activity (POD), and glutathione peroxidase (GPX) gene in tall fescue subjected to Cd and Pb treatment. Values are presented as a ratio to the untreated control, and the mean values marked with the same letter are not significantly different at *P*<0.05.

The expressions of *Cu/ZnSOD*, *FeSOD*, *POD*, and *GPX* were up-regulated at 24 h after Pb, Cd, or Pb+Cd application relative to the control (Fig 3). Plants treated with T4 exhibited higher expression level of *Cu/ZnSOD*, *FeSOD*, *POD*, and *GPX* when compared with T1. However, increased expressions of *Cu/Zn SOD*, POD were observed in T5 relative to T1, and no significant effects were detected in *FeSOD* and *GPX*. On the other hand, the expressions of *Cu/ZnSOD* and *GPX* were increased under both Pb and Cd treatments than single Cd^2+^ treatments regardless of Cd^2+^ application level. Notably, the expressions of *FeSOD* and *SOD* were increased in T4 compared with T1. However, with the same level of Cd, similar expressions of *FeSOD* and *SOD* were observed betweenT3 and T5.

## Discussion

Plants are usually exposed to many heavy metal elements simultaneously, and the integrated effects may be quite different from those ions acting alone [41]. The interactions between heavy metals are often complex. Therefore, the present study was undertaken to assess the interactive effects of Cd and Pb on seed germination, and to determine the contribution of anti-oxidative defense system in the expression of Cd and Pb toxicities during seedling growth. This would enrich our understanding of possible relationship of single Cd, Pb and their interaction (Cd+Pb) in tall fescue.

Germination has been recognized as the key step of plant life, and is more sensitive to heavy metal toxicity [42]. Our research has clearly illustrated that the presence of a low level of Cd (50 mg L^-1^) could alleviate the Pb toxicity on seed germination (Table 2). However, compared with single Cd^2+^ (50 mg L^-1^) treatment, this toxicity on germination was strengthened with the presence of Pb. This phenomenon can be attributed to the ions antagonism, suggesting that Cd and Pb influenced the uptake of each other [43]. With regard to seedlings, the NRT in both Cd+Pb treatments were lower than single Cd treatment at the same Cd^2+^ level. Compared with single Pb treatment, the NRT in both Cd+Pb treatment was decreased remarkably with the presence of 150 mM L^-1^ Cd^2+^. These results indicated the synergistic effects of Cd and Pb, and similar results were also reported on Cd-Zn [43]. However, Cd performed positive effects on VSGR under Pb stress suggesting the antagonistic action of Cd and Pb [44].

Electrolyte leakage and MDA are generally recognized as indicators of the degree of plant cell membrane damage, and reflected the ROS level under heavy metal stress [45]. Our results showed significant increment in EL and MDA content under single or combined Pb and Cd treatments due to the free radical-induced membrane damage [46]. Different responses were observed in MDA and EL when exposed to Pb and various Cd levels. As a result, plants exhibited lower EL and MDA content in Pb and low Cd treatment relative to single Pb treatment, but there were no significant decrements on MDA and EL under the presence of high Cd level. This phenomenon could be attributed to the detrimental damage of high Cd level on plant cell membrane [45]. Similar trends were also observed in soluble protein content, in our study.

Metal accumulation in plants differed greatly among organs or tissues [47]. Our research illustrated that more Cd and Pb were accumulated in roots than shoots (Table 4). The Pb content in shoots was decreased with the presence of Cd. In contrast, the Pb content in roots was increased with the presence of high level of Cd. Plants treated with both Pb and Cd exhibited lower translocation factor relative to single Pb treatment. The possible explanation for this phenomenon was that the root was the barrier for Pb translocation to the shoots, and the Pb may be precipitated as Pb-phosphate [3]. Meanwhile, with the presence of Pb^2+^, Cd content in shoots was increased and in contrary, Cd contents in roots were decreased. This may be attributed to the antagonistic and synergistic action of Cd and Pb [41]. Higher translocation factors were also assessed in both Cd and Pb treatments than single Cd treatment, suggesting that the plants resort to increased Cd^2+^ uptake to counteract Pb toxicity. Similar results were obtained by Chakravatry and Srivastava [48] who reported that linseed exhibited lower Cd uptake and higher Zn uptake under both Cd and Zn stress.

Excess production of ROS has been reported in many plants exposed to toxic levels of Cd and Pb, and ROS production has been recognized as one of the consequences of heavy metal toxicity [49, 50]. Meanwhile, plants are equipped with defense mechanism for repairing the ROS-induced damage, and antioxidant enzymes such as SOD, CAT, and POD activity which scavenge free radicals to prevent oxidant damage [51]. In the present study, the activity of SOD and POD were increased significantly under Cd, Pb, and Cd+Pb stress (Table 5), suggesting that plants adjusted their SOD and POD activity to protect membrane stability from heavy metal-induced oxidative damage. These results were consistent with the increased intensities of SOD and POD activity observed in tall fescue under Cd and Pb stress [52, 53]. However, the activity of CAT was decreased significantly when exposed to Cd, Pb, and Cd+Pb regimes. This phenomenon may be attributed to the carbonylation by non-protein thiols [54]. Similar results were illustrated by Srivastava et al. [50] who reported that decreased CAT activity was observed in rice seedlings under Cd and Pb stress. With regard to both Cd and Pb treatments, the activities of CAT and POD were increased relative to single Pb or low Cd treatment, suggesting the antagonistic action of the metals (Table 5). However, CAT activity in both Pb and high Cd treatments was lower than single Pb or Cd treatment due to the synergistic action. The increased CAT activity could be due to its role in elimination of rapidly produced H_2_O_2_ in both Pb or low Cd treatment, whereas under Pb and high Cd exposure, under high H_2_O_2_ production, its activity was not sufficient to eliminate H_2_O_2_, leading to excessive H_2_O_2_ build up in the plant tissues. In addition, by comparing expression levels of *Cu/ZnSOD*, *FeSOD*, *POD*, and *GPX* under single Cd, Pb, and combined Pb and Cd treatments (Fig 3), we concluded that antagonistic action occurred under both Pb and low level of Cd treatment. Low level of Cd application up-regulated all gene expressions that could produce more antioxidant enzymes to scavenge excessive ROS under Pb stress. Our results illustrated that over-expreesion of various SOD, POD, and GPX gene were corrected with the salt resistance due to the activated antioxidant system [21, 55].

In summary, the effects of Cd and Pb interaction on tall fescue were reflected at the seed germination; plant growth, physiological, antioxidant enzymes, and gene levels, and whether antagonistic action or synergistic action occurred depended on the level of metal ions. Antioxidant enzymes can be used as critical indicators of the responses to Cd and Pb interaction at an early stage of exposure in tall fescue, and gene expression profiles could provide more accurate description of the activation of those systems.
